# Surfing on Membrane Waves: Microvilli, Curved Membranes, and Immune Signaling

**DOI:** 10.3389/fimmu.2020.02187

**Published:** 2020-09-11

**Authors:** Ron Orbach, Xiaolei Su

**Affiliations:** ^1^Department of Cell Biology, Yale School of Medicine, New Haven, CT, United States; ^2^Yale Cancer Center, Yale University, New Haven, CT, United States

**Keywords:** microvilli, actin, membrane curvature, BAR protein, WASp, TCR, super-resolution microscopy, T-cell signaling

## Abstract

Microvilli are finger-like membrane protrusions, supported by the actin cytoskeleton, and found on almost all cell types. A growing body of evidence suggests that the dynamic lymphocyte microvilli, with their highly curved membranes, play an important role in signal transduction leading to immune responses. Nevertheless, challenges in modulating local membrane curvature and monitoring the high dynamicity of microvilli hampered the investigation of the curvature-generation mechanism and its functional consequences in signaling. These technical barriers have been partially overcome by recent advancements in adapted super-resolution microscopy. Here, we review the up-to-date progress in understanding the mechanisms and functional consequences of microvillus formation in T cell signaling. We discuss how the deformation of local membranes could potentially affect the organization of signaling proteins and their biochemical activities. We propose that curved membranes, together with the underlying cytoskeleton, shape microvilli into a unique compartment that sense and process signals leading to lymphocyte activation.

## Introduction

Sea looks calm miles away but wavy inches ahead; same applies to the plasma membrane. A variety of membrane protrusions have been identified on the cell surface, including microvilli, filopodia, lamellipodia, and cilia (see Table 1). Those structures play a classical function in sensing the environmental cues as well as facilitating cell migration. Meanwhile, accumulating evidence suggests that membrane protrusions also play an active role in regulating biochemical reactions that transduce membrane-proximal signaling (1–3), and dysregulation of their formation has been associated with diseases like Huntington’s disease, PAPA syndrome, Wiskott–Aldrich syndrome (WAS), and renal dysfunction (4–6).

**TABLE 1:** Comparison between common types of membrane protrusions.

	Microvilli	Filopodia	Lamellipodia	Cilia
Cell type	Most cells	Motile cells	Motile cells	All vertebrate cells, except for hematopoietic cells
Function	Signaling and motility	Sensory and guiding organelle	Motility	Signaling and motility
Diameter	50–350 nm	100–400 nm	Sheet-like structure	∼250 nm
Length	<4 μm	Up to 40 μm		1–10 μm
Cytoskeleton core structure	Actin	Actin	Actin	Microtubule
Organization	Parallel bundles	Parallel bundles	Branched network	Motile cilia: “9 + 2” Primary cilia: “9 + 0”
Other		Often emerge from lamellipodial sheets		Emerge from basal body

In the immune system, microvilli are among the most common types of membrane protrusions found on lymphocytes. Although they have been well-described by electron microscopy (EM) studies (7), the biochemical and signaling functions of microvilli remained neglected until recently. In this review, we discuss the potential of physical feature of microvilli in regulating chemical reactions that transduce membrane-proximal signaling. We also summarize the development of new techniques for imaging cell surface topography at high spatial or temporal resolutions, and for modulating membrane curvature in a precision manner, which could provide powerful tools for investigating the signaling function of microvilli.

## Formation of Dynamic Microvilli

**FIGURE 1:**
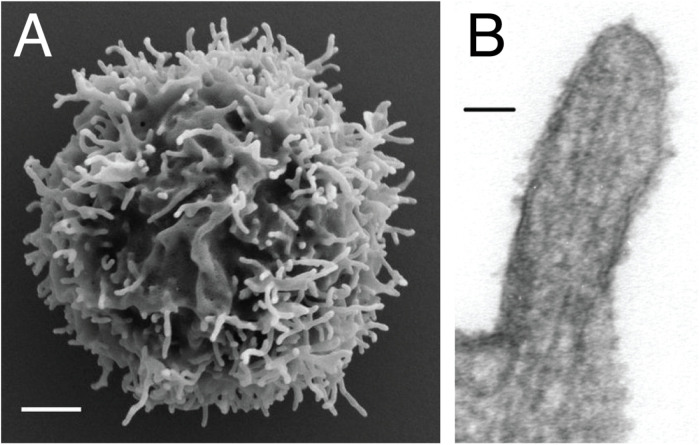
Microvilli decorating the plasma membrane of lymphocytes. **(A)** Scanning electron microscopy micrograph showing microvilli that protrude from the cell surface of resting peripheral blood human T cells. Scale bar: 1 μm. Reproduced from Jung et al. (19). Copyright 2016 National Academy of Sciences. **(B)** Transmission electron microscopy micrograph showing the parallel arrangement of F-actin within the microvilli of 300.19 cell (Abelson-transformed murine pre-B lymphoma) Scale bar: 50 nm. Republished with permission of ASH from Ref. (13).

Microvilli are thin finger-like membrane protrusions that are found on the surface of a wide variety of cell types (8), including intestinal epithelial cells (9), dendritic cells (10), and neurons (11). They are supported by actin filaments (F-actin) that are organized in parallel bundles of 10–30 filaments (12, 13), which resemble the actin network that constitutes filopodia (14). However, filopodia often protrude from the lamellipodial and lamellar actin network (15), while the microvilli actin network does not (16, 17). In the case of lymphocytes, EM studies showed the presence of microvilli on the surface of both T cells and B cells (18). The diameter of the microvilli ranges from 50 to 550 nm, as revealed by EM and fluorescence microscopy studies, while their length varies between 100 nm to several microns (Figure 1) (7, 13, 19, 20). Thus, microvilli dramatically increase the cell surface area, while having a negligible effect on the cytosolic volume. Furthermore, actin depolymerizing toxin Latrunculin A (LatA) eliminates most microvilli within 1 min in a reversible manner, suggesting that microvilli are highly dynamic structures (13). Recent technological advances in lattice light-sheet microscopy allow 3D real-time tracing of such dynamic microvilli (20). It was discovered that microvilli move laterally on the plasma membrane and survey antigen-presenting cells (APCs) within 1 min, which is, coincidently or not, the half-life of T cell–APC contact duration *in vivo* (20). Therefore, the dynamics of microvilli fits well into their function in searching antigens.

Despite a handful of studies on microvilli morphology, our knowledge on the regulatory mechanism of microvilli size, structure, and dynamics is still limited. Evidently, their fate following the formation of immunological synapse is still a matter of debate. Cai et al. (20) demonstrated that there is no change in the microvilli density before and after the immunological synapse is formed. In contrast, Kim et al. (21) showed that at an early stage of synapse development microvilli polarize toward the synapse, but as the synapse matures, most of the microvilli disappear. The later was further supported by a recent study by Ghosh et al. (22), showing the loss of the microvilli after T cell receptor (TCR) stimulation.

The microvilli are also regulated by cytokines and chemokines. Westerberg et al. (23) found that CD40 antibody together with IL-4 induces microvilli on the surface of B cells. On the other hand, the chemokines stromal derived factor 1α (SDF-1α) and B lymphocyte chemokine (BLC) induce resorption of microvilli (24, 25), which promotes B cell homing by transition from rolling adhesion to integrin-mediated adhesion. Not surprisingly, members of the ezrin-radixin-moesin (ERM) family, which link the cortical F-actin cytoskeleton to the plasma membrane, were found to regulate microvilli assembly, namely, dephosphorylation of ERM proteins (ezrin, T567; radixin, T564, moesin, T558), induced by chemokines, results in resorption of microvilli within a few seconds (25, 26). Because ERM dephosphorylation can be triggered by TCR activation (27), ERM could mediate TCR-induced microvilli resorption (22).

Interestingly, changes in the microvilli shape and density are also linked to several diseases. Uneven distribution of long microvilli was observed on B cells from hairy cell leukemia patients (28–31). Moreover, changes in microvilli morphology were observed in WAS, a severe immunodeficiency disorder that is caused by defective or missing Wiskott–Aldrich syndrome protein (WASp). WASp activates Arp2/3 complex by inducing a conformational change of Arp2/3 and by delivering the first actin monomer of the nascent filament (32–36). On the other hand, WASp could directly promote actin polymerization independently of Arp2/3 (37). It has been demonstrated that lymphocytes derived from patients with WAS, either in resting or activated states, exhibit various microvillar morphological abnormalities. These abnormalities include a decrease in microvilli density and length, as well as formation of dysmorphic structures (5, 24, 38–42). However, knockdown of Arp2 in Jurkat T cells caused no significant effect on microvilli assembly (43), which suggests that WASp might regulate microvilli formation independent of Arp2/3. Meanwhile, further studies are required to confirm the Arp2 phenotype in primary T cells.

## Can Microvilli Serve as a Signaling Center?

The notion that microvilli could serve as a signaling center was primed by studies showing that certain signaling proteins are enriched in microvilli. Immunogold EM studies demonstrated the enrichment of various receptors and adhesion molecules on the microvilli, including insulin receptors, selectin, integrin, and the T cell co-receptor CD4 (44–49). Mass-spectrometry analysis was also implemented to compare the protein composition between isolated microvilli and whole cell, from both human peripheral blood T-lymphocytes and a mouse pre-B lymphocyte line (50). It revealed that microvilli are enriched of GTP-binding proteins, cytoskeletal proteins, and transmembrane proteins as compared to the cell body (after removing the nucleus). This study provides the first global mapping of the microvilli proteome. However, it should be noted that using the cell body as a control could lead to the identification of membrane-associated proteins rather than microvilli-specific proteins, because the surface-to-volume ratio is much higher in microvilli as compared to the cytoplasm. It should be also noted that the identification of membrane proteins by mass-spectrometry remains as a challenge because of the proteins limited solubility in aqueous buffer (51). Therefore, certain hits might be missing in the dataset. Thus, orthogonal approaches will be needed to verify the microvilli-enriched proteins.

For many years the dynamic nature of microvilli together with their small dimensions have hindered the structural and functional characterization of microvilli. Although EM provides high spatial resolutions, understanding the signaling function of microvilli requires characterizing microvilli morphology and signaling protein localization with high temporal resolutions. The advancement of fluorescence microscopy techniques in the past decades has enabled an investigation of membrane morphology and protein localization in microvilli at either high temporal or spatial resolutions, though the combination of both is still technically challenging (19–22, 52, 53).

**FIGURE 2:**
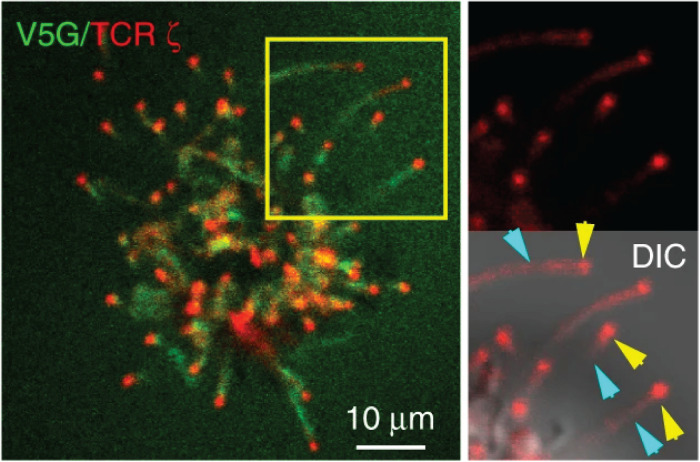
TCR localizes to the microvilli tip. Jurkat T cells expressing GFP-V5G and TCRζ-tdTomato were imaged at the terminal stage of T cell activation. Reproduced from Kim et al. (21) licensed under Creative Commons (CC BY 4.0).

The Haran group comprehensively characterized the localization of TCR signaling proteins on the microvilli by a unique imaging technique that allows accurate mapping of membrane protein localization. By combining variable-angle total internal reflection microscopy and stochastic localization nanoscopy, the authors reconstructed 3D topographical maps of T cells (19, 22). They have shown that TCR, co-receptor CD4, kinase Lck, adaptor LAT, and adhesion receptor CD2 are highly enriched in the microvilli. On the sub-microvilli scale, the Jun group, using Total Internal Reflection Fluorescence (TIRF) microscopy, showed that TCR is specifically enriched on the microvilli tip (Figure 2) (21). Thus, the tip localization of TCR could promote searching of antigens and establishing contacts with APCs (20). The Haran group also found that treatment with LatA or expressing a dominant negative form of ezrin, both of which reduce microvilli, leads to a random distribution of TCR throughout the plasma membrane. Intriguingly, the authors showed that following TCR stimulation, T cells lose their microvilli, which consequently leads to an even distribution of TCRαβ throughout the plasma membrane (22). This result suggests that microvilli-dependent TCR enrichment could be regulated by TCR triggering.

The “kinetic-segregation” model serves as one of the prevalent mechanisms explaining TCR triggering (54–57). A key part to this model is the segregation of the large tyrosine phosphatase CD45 from the TCR-pMHC contact zone. Therefore, multiple groups have investigated the localization of CD45 in the context of microvilli and showed that the segregation between TCR and CD45 occurs a few seconds after contacts are established (58–61). Interestingly, although it has been assumed and supported by experimental data that CD45 is evenly distributed on the cell surface in resting T cells (19, 60), a new study revealed, using expansion microscopy, that CD45 is excluded from the microvilli tip even before contacts are established with APC (53). These discrepancies could be caused by differences in T cell subtypes, activation methods, and resolution of individual imaging techniques.

**FIGURE 3:**
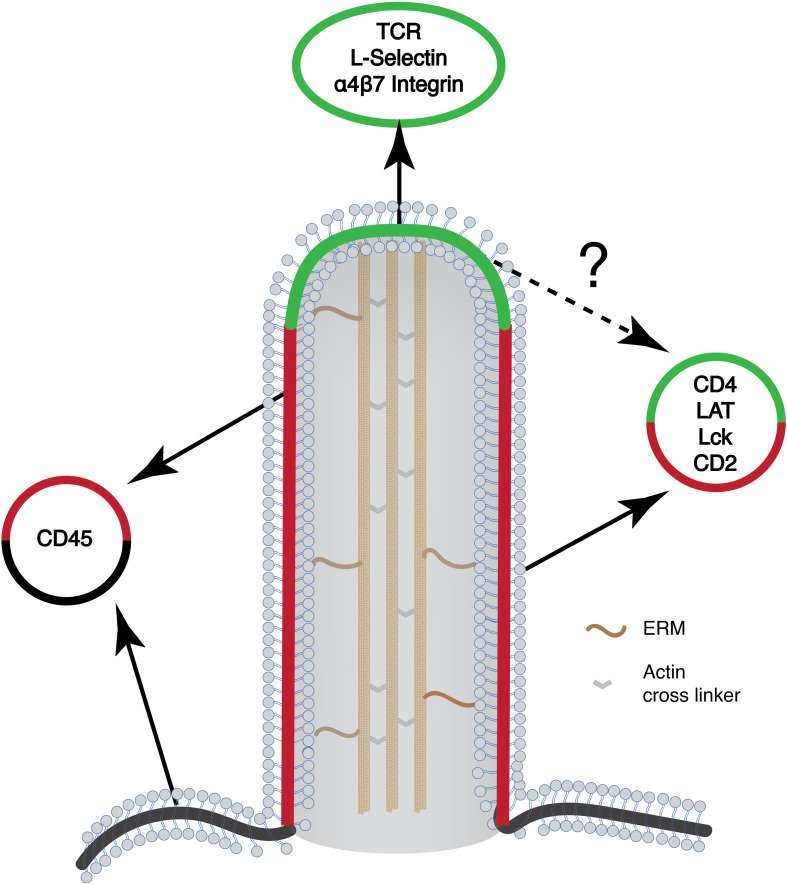
Schematic of the microvilli and the organization of different signaling proteins on the microvilli. Many of the signaling molecules that are involved in T cell activation preferentially localize to the microvilli. Yet, their organization within the microvilli is not known (marked in question mark). Green, microvilli tip region; red, microvilli body; black, plasma membrane.

Summarizing localization studies above, key components mediating TCR-proximal signaling reside in microvilli. These include TCR itself, kinase Lck, and adaptor LAT. It is expected that cytosolic proteins that are associated with these membrane proteins, including ZAP70, Grb2, Sos1, PLCγ1, Gads, and SLP76, are likely to be enriched in microvilli as well. The physical proximity of these molecules could increase the rate of chemical reactions and efficiency of signal transduction. Microvilli, therefore, could serve as a compartment to enrich signaling proteins to promote TCR signaling (Figure 3). In this regard, T cell microclusters are another entity that has been proposed for promoting TCR signaling (62, 63). Because both microvilli-localized proteins and T cell microcluster components display a puncta-like structure on the cell membrane, it raises an interesting question on the relationship between the two. Current evidence suggests that these two entities are different but related structures. The microvilli-enriched proteins are mostly characterized in resting T cells and microvilli disappear, at least in some studies, after TCR activation (22). In contrast, T cell microclusters are formed after TCR activation (62, 63). They also displayed limited mobility as compared to the highly mobile microvilli (20). Meanwhile, the “pre-enrichment” of signaling components in microvilli could facilitate T microcluster formation upon TCR activation.

Besides a potential signaling function in T cells, microvilli have also been proposed by the Jun Lab to serve as precursors for generating TCR-enriched extracellular vesicles (or synaptosomes) that activate dendritic cells (21). It remains to be determined whether the synaptosomes are similar or different to other TCR-enriched microvesicles (synaptic ectosomes) that were described by the Dustin Lab (64). Ectosomes are generated by the ESCRT complex, of which TSG101 facilitates the sorting of TCR into the ectosomes whereas Vps4 facilitates the scission of ectosomes from the plasma membrane. Interestingly, CD40L, a key effector delivered by helper T cells to activate APC, is also enriched in the microvilli and ectosomes, though CD40L is spatially segregated from TCR in ectosomes (65). This probably suggests that TCR and CD40L are independently sorted into microvilli, whereas the exact mechanism needs to be determined. It also remains as an intriguing question on how many microvilli and their associated TCRs end up in synaptosomes or ectosomes. Is ESCRT, a general membrane-shaping machinery, involved in microvilli dynamics regulation and resorption? Answering these questions will help to generate a complete picture of the microvilli life cycle during T cell activation (65).

## Mechanisms for Inducing Membrane Curvature and Protein Enrichment

Highly curved membranes represent a unique feature of microvilli, which could also serve as a platform for enriching proteins in microvilli. Several mechanisms have been proposed for generating curved membranes. Polymerization of actin filaments drives membrane protrusion; in parallel, membrane-associated proteins can also induce membrane curvature by the insertion of conical transmembrane proteins or hydrophobic protein domains into the membrane (66, 67). Intriguingly, intrinsically disordered domains, when attached to membranes, can drive membrane protrusion either with a positive or negative curvature (68–70).

The Bin-Amphiphysin-Rvs (BAR) superfamily is a key player involved in regulation, formation, and detection of cell membrane curvature (71). In this superfamily, the N-BAR and the F-BAR are associated with positive curvatures (e.g., membrane invagination or endocytic pits). In contrast, the I-BAR subfamily of proteins is associated with negative curvatures as found in various membrane protrusions (72). Many members of the BAR superfamily contain the structurally conserved SH2 or SH3 domains that recruit their binding partners to the curved membranes (73). One particular interesting example is the I-BAR protein IRSp53 (also known as BAIAP2) that binds cytoskeletal effectors such as N-WASP through its SH3 domain (74). In an *in vitro* biochemical assay, IRSp53 induces tubular membrane protrusions with similar dimensions of microvilli (75). IRSp53 is localized to filopodia when ectopically expressed in neuronal NSC34 cells (76) and regulates filopodia dynamics (77). Interestingly, IRSp53 is also expressed in T cells (78), raising its possible role in regulating microvilli formation. Surprisingly, the N-BAR protein sorting nexin 9 (SNX9), which is expected to recognize positive curvatures, is involved the biogenesis of filopodia (79). In a cell-free system for reconstituting actin bundles of filopodia (80), immunodepletion of SNX9 resulted in shorter actin bundles. Moreover, SNX9 localizes to the filopodial tip and shaft in RPE-1 cells (79). The positive role of SNX9 in filopodia formation is dependent on its activity in stimulating N-WASp and Arp2/3 (81), which probably overrides the curvature-sensing function of the N-BAR domain. In terms of the function of SNX9 in T cells, SNX9 was found to interact with WASp, p85, and CD28 to form a signaling complex on endocytic vesicles when T cells are activated by soluble CD3/CD28 antibodies (82). It remains to be determined if SNX9 can regulate microvilli when T cells are activated by surface or bilayer-presented stimuli because stimuli with a physical support could cause different outcomes as compared to those in a soluble format. Previous reports showed that TCR is internalized through endocytosis when T cells are treated with soluble MHC tetramer (83), whereas TCR is sorted into extracellular microvesicles when T cells are activated by supported lipid bilayer (SLB)-presented pMHC (64).

While the BAR proteins sense membrane curvature within the nanometer scale, there are proteins that can also sense a larger length scale. Septins and stage V sporulation protein M (SpoVM) sense positive micron-scale curvatures (84, 85). It was suggested that these nanometer-sized proteins sense micron-scale curvature by polymerization into micro-scale filaments. In contrast, the protein machinery that directly senses micron- or submicron-scale negative curvatures remains to be determined.

What is the cellular function of membrane deformation? Various studies have highlighted the role of membrane curvature in regulating sorting of transmembrane proteins (86–89). Using patterned nanostructure surfaces, Zhao et al. (90) found that the protein machinery mediating clathrin-mediated endocytosis prefers a positive curvature with a radius below 200 nm. Liang et al. (91) discovered that small GTPase Ras senses membrane curvatures in an isoform-dependent manner. One isoform binds to membranes with low curvatures, whereas the other binds to membranes with high curvatures. The effect of local membrane curvature may also influence cell polarization. For many years it had been assumed that cell polarization is induced exclusively by a gradient of a chemoattractant. A recent report revealed that chemical signaling is not sufficient for inducing cell polarization of neutrophils and CD8^+^ T cells (92). Instead, the authors found that polarization initiates with the formation of curved membranes, which recruits BAR domain protein SRGAP2, activates PI4KA, and results in PtdIns4P polarization. Furthermore, to understand the mechanism by which membrane curvature affects actin-dependent processes, such as endocytosis, focal adhesion maturation, and stress fiber organization, Lou et al. (93) have used patterned nanostructure surfaces to study actin rearrangement. Intriguingly, the authors found that the actin nucleator Arp2/3 and its regulators N-WASP and cortactin are recruited by BAR proteins to membranes with positive curvatures (with radii <200 nm). Consequently, branched actin networks assemble around curved membranes, depleting the monomeric actin pool for assembling stress fibers and mature focal adhesions. Interestingly, members of the formin family, which promote the polymerization of linear F-actin, showed no preferential localization to curved membranes.

The lipid composition of the plasma membrane also influences membrane geometry and protein localization. The size of the lipid headgroups, their charge, as well as the saturation state of acyl chains determines lipid shapes, and consequently the local membrane curvature (66, 94). Lipids with small headgroups such as cardiolipin, phosphatidylethanolamine, ceramide, diacylglycerol, and phosphatic acid induce negative membrane curvatures, whereas lipids with large headgroups like lysophosphatidylcholine and phosphatidylinositol phosphate induce positive curvatures (95). Some of these lipids can also recruit proteins to the membrane. For example, the negatively charged lipids phosphatidylserine and phosphatidylinositol 4,5-bisphosphate recruit positively charged proteins by electrostatic interactions (96–98). Sphingomyelin was found to selectively localize to the microvilli of epithelial cells and to induce microvilli formation through the indirect recruitment of ERM proteins (99). Whether this is also the case in lymphocytes and what the role of sphingomyelinase is in regulating microvilli formation need to be further explored. On the other hand, lipids can mediate the exclusion of proteins from curved membranes. A recent intriguing study from Jung et al. showed that CD45 is excluded out of the tip of microvilli in a cholesterol-dependent manner (53). Although the localization of cholesterol needs to be determined in the context of microvilli, cholesterol was previously reported to be enriched in the negative curved membranes in vitro or in silico (100, 101), where it, together with sphingomyelin, also thickens the membrane (102, 103). The thickened membrane caused by the accumulation of cholesterol was suggested to exclude CD45 of which the transmembrane domain is not long enough to be integrated into the thickened membrane. Depletion of cholesterol by cyclodextrin reduced the exclusion of CD45 from the tip, accompanied by a decrease in the membrane thickness and number of microvilli (53, 104). Besides the contribution from individual lipids, membrane tension, by serving as a physical barrier, can antagonize actin based-protrusion (105).

The composition and organization of the glycocalyx layer, which covers the outer leaflet of the plasma membrane, also contributes to cell morphology and membrane protrusions (106). Mucins are flexible transmembrane glycoprotein polymers within the glycocalyx that are enriched on the surface of many membrane protrusions, such as epithelial microvilli (107). A recent study has demonstrated the role of the mucins in generating forces driving the tubularization of the plasma membrane (108). In contrast, rigid glycoproteins have not shown similar phenomenon as the mucins. In the case of T cells, many cell surface proteins are highly glycosylated, among which CD43 and CD45 are the most abundant glycoproteins (109, 110). Notably, different isoforms of CD45 are expressed at different T cell development stages, and these isoforms differ significantly in their extracellular domain sizes (58, 111). It remains as an interesting question whether these isoforms contribute differently to microvilli formation.

## Approaches to Manipulate Microvilli and Membrane Curvature

Investigation of the microvilli function can be extremely challenging due to limited tools to specifically manipulate them in cells without perturbing other actin-based processes. Moreover, their small dimensions (*r* < 200 nm) and unique architecture (i.e., negative membrane curvature viewed from inside of microvilli) hamper the application of traditional *in vitro* reconstitution approach to the study of microvilli. Nevertheless, methods for studying filopodia, which present similar structural properties as the microvilli, as well as other recent technological advances that enable the accurate shaping of membranes, can be implemented to interrogate microvilli.

### Genetic Approaches

In microvilli the actin filaments are organized in parallel bundles (13). Various crosslinkers such as fascin, fimbrin, and espin promote the formation of actin bundles (112–115). While direct evidence is required, these actin crosslinkers can be attractive targets for specifically modulating microvilli shape and density, as demonstrated in filopodia (113). The ERM family, another component involved in microvilli formation, plays a major role in connecting actin cytoskeleton to the cell membrane. Overexpressing a dominant-negative form of ezrin dramatically reduces microvilli formation (24). Yet, a recent finding suggests that the enrichment of ezrin around membrane protrusions is facilitated by I-BAR-domain proteins (116).

### Pharmacological Approaches

Complementing genetic manipulations, pharmacological treatments perturbing membrane composition or cytoskeleton can modulate microvilli in a rapid fashion. Greicius et al. (104) reported a decrease in microvilli density by depleting cholesterol using cyclodextrin. A similar effect could be achieved by using the actin depolymerizing toxin LatA (13, 19). However, both drugs are expected to affect the whole membrane structure, actin network, and surface presence of many signaling proteins, all of which may complicate the interpretation of the results regarding microvilli-specific functions. Recently, an inhibitor of the crosslinker protein fascin has been identified, which could be potentially used to manipulate microvilli. This inhibitor blocks the activity of fascin to bundle actin filaments *in vitro*, and filopodial formation in multiple cell lines. Furthermore, it blocks cancer cell metastasis, potentially by inhibiting filopodia formation (117).

### Physical Approaches

**FIGURE 4:**
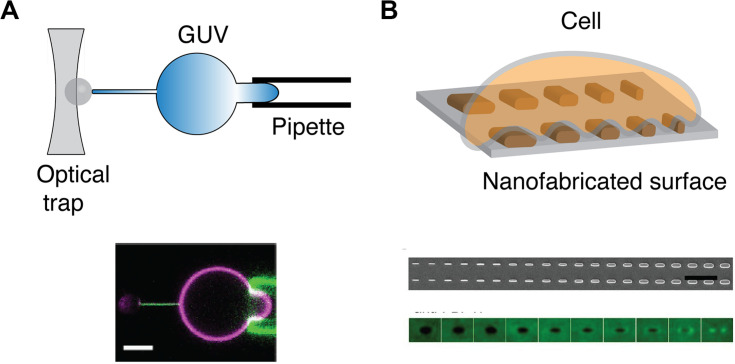
Methods to physically manipulate membrane curvature. **(A)** Schematic of optical trap that is used to pull a thin nanotube from GUV held by a pipette (top). Confocal microscopy reveals that GFP-IRSp53 BAR protein localizes to nanotube pulled from GUV (magenta) that is held by a pipette (bottom). Scale bar: 5 μm. Reproduced from Prévost et al. (118) licensed under Creative Commons (CC BY 4.0). **(B)** Schematic of a cell on a nanofabricated surface with structures of different radii (top). Scanning electron microscopy microfabricated of nanofabricated chip with a gradient nanobar array with a variable width from 100 to 1,000 nm (100 nm increment; bar length: 2 μm) (middle). The averaged nanobar images of anti-FBP17 immunostaining for 10 different nanobar widths. FBP17 localizes to positively curved structures with a width <400 nm (bottom). Reproduced from (93). Copyright 2019 National Academy of Sciences.

*In vitro* assays have been developed to isolate the effect of membrane curvature from complex cellular environment. Yet, while methods to generate positive membrane curvature are well established, it is not the case with negative membrane curvatures, especially in the range of microvillus sizes (*r* < 200 nm). In one approach, a giant unilamellar vesicle (GUV) is held by a micropipette at one side and pulled, on the other side, by a polystyrene bead holding by optical traps (Figure 4A). A membrane nanotube can be generated with controlled radii, ranging from 7 to 100 nm, by adjusting the micropipette pressure (118). Similarly, an optical trap has been used to pull short tethers (*r* < 100 nm) from the cell membrane (119). Meanwhile, these manipulations are technically challenging and may be time-consuming. GUVs are also sensitive to osmotic changes and therefore can bring difficulty to long-duration experiments. Alternative approaches to study negative curvature employ substrates that serve as a mold to induce membrane curvature on artificial membranes and cells (Figure 4B). Focused ion beam has been applied to etch an array of invaginations with a radius of 100 nm on a glass surface (120). The fabricated substrate can then be covered with SLB to mimic the highly curved membranes in microvilli. Another promising means to induce membrane curvature is by using nanofabricated substrates (90, 121). Yet, the diffusion of membrane components may be affected by the substrate (122, 123), which should be carefully evaluated beforehand.

## Outlook

Looking forward, significant questions remain to be addressed in terms of the mechanism and signaling function of T cell microvilli:

(1) What are the mechanisms that regulate microvilli formation and dynamics? Besides identifying the key protein and lipid components regulating microvilli, it will also be necessary to understand the relationship between microvilli and other membrane structures, for example, the recently identified CD2 Corolla which seems to be devoid of microvilli (124).
(2) How do microvilli regulate the localization and oligomerization state of proteins and lipids? Phase separation, or the formation of liquid-like microclusters, emerges as a new principle in regulating TCR signaling (125, 126). The unique membrane topology in microvilli could play an important role in regulating the assembly of signaling microclusters. On the other hand, microvilli could bring proteins physically close even if there are no direct interactions between those proteins. Future co-localization studies should be best performed in the context of microvilli to understand the exact nature of the entities that are examined. In addition, many other tiny and transient proteo-lipid nanodomains have been identified on the plasma membrane (127–130). It remains as an open question on the relationship between these structures and the microvilli-localized proteins.
(3) How do microvilli modulate chemical reactions? The src-family kinase Lck has been shown to be enriched in microvilli (22). Future studies are expected to reveal the microvilli localization or even sub-microvilli localization (tip, side, or base) of other enzymes in the TCR signaling (e.g., PLCγ1, ZAP70, SHP1, CBL), together with their corresponding substrates. Elegant reconstitution approaches will be needed to recapitulate the essential physical and chemical environment of microvilli to understand how curved membranes in microvilli affect the specific activity of kinases, phosphatases, and lipases.
(4) How do microvilli regulate TCR signal transduction? As protrusive structures that search the surrounding space and make contacts with the APCs, microvilli are constantly experiencing mechanical forces from the environment (131). These forces may be involved in the regulation of cell recognition and calcium flux, as found in the microvillar photoreceptor cells (132). Moreover, TCRs on the tip of the microvilli receive stimuli from APCs. Meanwhile, what happens after antigen recognition remains unclear. How is signal transduced from the tip of microvilli to the cell body? Do microvilli participate in kinetic proof-reading since microvilli are enriched with proteins mediating multiple steps along the TCR pathway? Do microvilli serve as a signaling unit that integrates signals from TCR and co-receptors first before sending them to the cell body, or are different signals transduced individually across the microvilli? With these questions being addressed, the current map of T cell membrane signaling is likely to be significantly expanded from a 2D surface to a 3D world.

## Author Contributions

RO and XS conceived and wrote the manuscript. Both authors contributed to the article and approved the submitted version.

## Conflict of Interest

The authors declare that the research was conducted in the absence of any commercial or financial relationships that could be construed as a potential conflict of interest.
